# *N*-Glycosylation Profiling of Human Blood in Type 2 Diabetes by Capillary Electrophoresis: A Preliminary Study

**DOI:** 10.3390/molecules26216399

**Published:** 2021-10-22

**Authors:** Rebeka Torok, Klaudia Horompoly, Marton Szigeti, Andras Guttman, Marta Vitai, Laszlo Koranyi, Gabor Jarvas

**Affiliations:** 1 Translational Glycomics Research Group, Research Institute of Biomolecular and Chemical Engineering, University of Pannonia, 8200 Veszprem, Hungary; torokrebeka@gmail.com (R.T.); horompoly.klaudia@gmail.com (K.H.); szigeti.marton@gmail.com (M.S.); guttman@mik.uni-pannon.hu (A.G.); 2 Horvath Csaba Memorial Laboratory of Bioseparation Sciences, Research Center for Molecular Medicine, Faculty of Medicine, Doctoral School of Molecular Medicine, University of Debrecen, 4032 Debrecen, Hungary; 3 DRC Drug Research Center Ltd., 8230 Balatonfured, Hungary; vitai.marta@gmail.com (M.V.); laszlo.koranyi@drc.hu (L.K.)

**Keywords:** *N*-glycan, type 2 diabetes, capillary electrophoresis, biomarker, blood collecting tubes

## Abstract

Currently, diagnosing type 2 diabetes (T2D) is a great challenge. Thus, there is a need to find rapid, simple, and reliable analytical methods that can detect the disease at an early stage. The aim of this work was to shed light on the importance of sample collection options, sample preparation conditions, and the applied capillary electrophoresis bioanalytical technique, for a high-resolution determination of the *N*-glycan profile in human blood samples of patients with type 2 diabetes (T2D). To achieve the profile information of these complex oligosaccharides, linked by asparagine to hIgG in the blood, the glycoproteins of the samples needed to be cleaved, labelled, and purified with sufficient yield and selectivity. The resulting samples were analyzed by capillary electrophoresis, with laser-induced fluorescence detection. After separation parameter optimization, the capillary electrophoresis technique was implemented for efficient *N*-glycan profiling of whole blood samples from the diabetic patients. Our results revealed that there were subtle differences between the *N*-glycan profiles of the diabetic and control samples; in particular, two *N*-glycan structures were identified as potential glycobiomarkers that could reveal significant changes between the untreated/treated type 2 diabetic and control samples. By analyzing the resulting oligosaccharide profiles, clinically relevant information was obtained, revealing the differences between the untreated and HMG-CoA reductase-inhibitor-treated diabetic patients on changes in the *N*-glycan profile in the blood. In addition, the information from specific IgG *N*-glycosylation profiles in T2D could shed light on underlying inflammatory pathophysiological processes and lead to drug targets.

## 1. Introduction

Type 2 diabetes (T2D) is an extremely challenging health issue of the 21st century [1,2]. It is a multifactorial disease that results from a combination of environmental and genetic factors. Although many environmental and genetic risk factors have already been identified, the underlying mechanisms of the disease remain largely unknown. The genetic factors identified so far only explain up to 5–10% of the disease risk [3]. It is likely that epigenetic and post-transcriptional modifications of glycoproteins play a substantial role in the disease pathophysiology [4].

The *N*-glycosylation of proteins is an important post-translational modification, representing a good source of biomarker discoveries [5]. Global *N*-glycan profiling has recently gained high importance for both fundamental biomedical and applied clinical research, as well as for diagnostic purposes, e.g., in the cases of diabetes, chronic obstructive pulmonary disease (COPD), and prostate cancer [6,7,8]. Due to the recent advances in bioseparation methods and the associated software tools, *N*-glycosylation has been targeted in the search for disease biomarkers for early diagnosis and patient stratification. Biofluids such as saliva, serum, or plasma are great sample sources in this regard, as they are easily accessible and can provide the relevant glycosylation information. The increasing knowledge of the role of *N*-glycans in cancer has suggested that glycobiomarkers could be useful in new therapeutic and diagnostic approaches in other diseases as well [9]. Currently, the most common source used in biomarker research is blood, because it contains very high numbers of lipids, proteins with associated glycans, DNA, RNA, and miRNA, to mention the most important ones. The high-abundant proteins in blood include Immunoglobulin G (85%), Immunoglobulin A, transferrin, haptoglobin, and α-1-antitrypsin. All of the above are glycosylated, and their attached carbohydrate structures can be analyzed by liquid chromatography (LC) and its hyphenation with mass spectrometry (LC-MS), capillary electrophoresis with laser-induced fluorescence detection (CE-LIF) and capillary electrophoresis–mass spectrometry (CE-MS). Thus, these glycan biomarkers show great potential in the growth of chronic-disease-related glycomics and glycoproteomics, which are the next great challenge after genomics and proteomics [10]. The changes in IgG *N*-glycosylation have reportedly been associated with various diseases, including rheumatoid arthritis, cancers, many chronic-metabolic diseases, and some inflammatory diseases, such as ulcerative colitis, type 1 diabetes, and T2D [2,11]. Therefore, these complex *N*-glycan structures may be altered by chronic inflammations. Several publications have reported that the presence of bisecting *N*-acetylglucosamine (GlcNAc) [12], and the lack of core fucosylation [13], increase the antibody-dependent cell-mediated cytotoxicity (ADCC) effect of IgG, whereas the decreased percentage of galactosylation could elevate the complement-dependent cytotoxicity, thus strengthening its proinflammatory function. Several IgG glycans and traits are firmly associated with T2D, reflecting a pro-inflammatory and biological-aged state, such as a decrease in galactosylation and sialyation, an increase in fucosylated structures with bisecting GlcNAc, and a decrease in fucosylated structures without bisecting GlcNAc [2,10].

Generally, in the case of the total plasma *N*-glycome, gender was a significant predictor in the IgG glycosylation, although only for some structures containing bisecting GlcNAc. It was also assumed that the heritability of the *N*-glycan profile was generally between 25% and 45%, indicating that a significant part of the variability of IgG glycosylation can be explained by genetic polymorphisms, and that gender is not an important predictor for IgG glycans [14]. Quite the opposite, the age of the participating members was a determining factor in the case of *N*-glycosylation [15,16]. In these studies, the individual’s age was associated with a significant decrease in galactose, and an increase in bisecting GlcNAc, whereas the other functional elements of IgG glycosylation did not change much with age, such as sialylation. Sialylation appeared to be the most endogenously defined glycosylation feature, with up to 60% of its variance explained by heritability. Age had very little effect on the extent of sialylation, whereas gender was nearly irrelevant [16]. Furthermore, it was also shown that the obtained *N*-glycan profiles were stable over a 6-month period in T2 diabetic patients, and could be used to monitor the biochemical changes in relation to T2D comorbidities [17].

Capillary electrophoresis offers high separation efficiency, rapid analysis time, simple method development, low sample and solvent consumption, and a cost-effective platform for the *N*-glycan characterization. The CE separation allows high sensitivity and high-resolution separation at the same time. Furthermore, the compatibility of capillary electrophoresis with various detectors makes it suitable for: the analysis of biological macromolecules; chiral compounds [18]; vitamins [19]; pesticides [19]; inorganic ions [20]; organic acids [21]; peptides; proteins [22]; carbohydrates [23]; oligonucleotides [24]; DNA fragments [25]; and the separation and qualitative determination of whole cells viruses, and anions [26]. The separation conditions must be optimal in order to achieve the appropriate results, including temperature, buffer pH and ionic strength [27]. Commonly, ultra-performance liquid chromatography (UPLC) is used to analyze the *N*-glycan profile of type 2 diabetic samples [17]. In the literature, the UPLC analysis revealed the increase in the complex oligosaccharides (biantennary and high mannose) and the decrease in the rate of fucosylation of digalactosylated glycans [2,10]. In addition, the rate of galactosylation and sialylation in fucosylated structures varied depending on the bounding of the bisecting *N*-acetil-glucosamin [10]. Both the CE and UPLC techniques are high-resolution methods that produce a complementary fingerprint of the glycoform distribution, and allow for quantitative and reproducible determinations [18]. However, the obtained profiles were significantly different, due to the applied diverse sample-preparation chemistries and separation method orthogonality [28]. In contrast to the UPLC, the advantages of CE in analyzing the *N*-glycan profiles of T2D include the fast analysis time, very small sample need, and complementary information. However, due to the addition of salts in the reaction buffer, the samples require an extensive clean up [29].

In this study, capillary electrophoresis with high-sensitivity fluorescence detection was used for the analysis of *N*-glycans from the whole blood of T2D patients. First, the effects of sample re-freezing and the type of the blood-collection tubes were investigated on *N*-glycosylation profiles of whole blood, and serum samples from healthy and diabetic patients. It was found that two potential glycan biomarkers could carry diagnostic information in the case of T2D.

## 2. Results

The research objectives of this work included the application of capillary electrophoresis-based high-throughput *N*-glycan profiling, for the analysis of type 2 diabetic blood samples, including sample preparation and separation with high sensitivity. Our major goal was to highlight some important aspects of the sample collection and preparation conditions that significantly alter the obtained results. We also comparatively investigated T2D blood and serum samples with control-pooled blood and serum samples from healthy volunteers.

**Figure 1:**
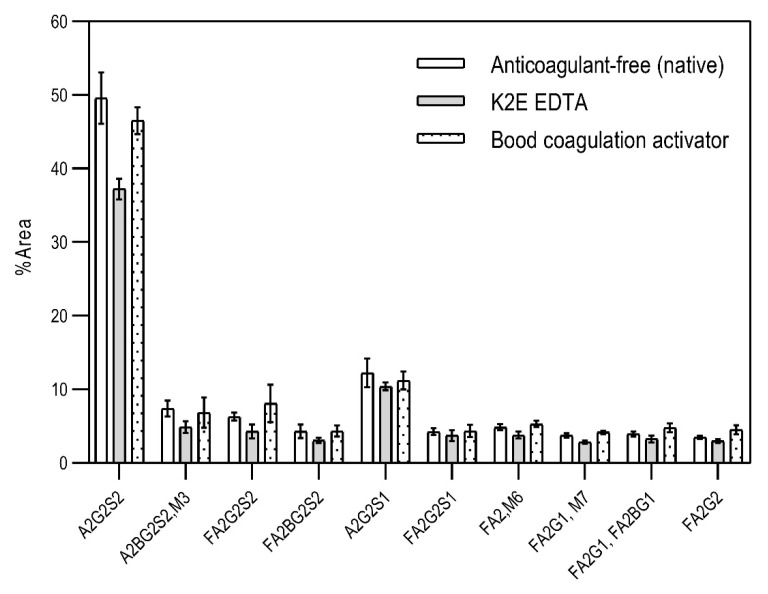
The effect of different types of blood-collection tubes on the resulting *N*-glycan profiles of whole blood (healthy) after two months with 5 intermittent re-freezing cycles. Separation conditions: 30 cm total (20 cm effective) length, 50 µm i.d. bare fused silica capillary at 20 °C, HR-NCHO separation gel buffer, two-stage injection: 5.0 psi for 5 s water plug → 6.0 kV for 3 s sample.

First, the effects of the blood-collection parameters, sample handling, and sample-storage conditions were investigated on the resulting *N*-glycan profile. The initial goal of this analytical approach was to be introduced in a regular hospital patient-care system. Thus, different blood-collection tubes—such as (1) anticoagulant-free, (2) K2E EDTA, and (3) blood-coagulation activator—were tested first and compared for their effect on the blood *N*-glycosylation profile. In addition, the blood samples were exposed to non-ideal sample storage and handling conditions. The samples were thawed and re-frozen five times in a 2-month period (approximately every two weeks) to be able to check the stability of the *N*-glycan structures in the different blood-collection tubes. All measurements were performed in triplicates. The sample-preparation parameters were optimized: (1) all samples were drawn through the septum of the collection tube in order to preserve the original sample; (2) all samples (serum and whole blood) were diluted 50× with HPLC-grade water; and (3) APTS labeling was set at 37 °C overnight, with the lid open (evaporative labeling) in order to assure full derivatization. The blood samples were prepared and analyzed by CE–LIF at each thaw cycle, and the resulting % areas of the major glycan peaks were averaged for the entire series of measurements, including the analysis of the initial fresh sample (Figure 1). The error bars in Figure 1 represent the standard deviation of the mean of all the measurements.

The results in Figure 1 show the major differences in the peak distribution between the application of the different blood-collection tubes. However, in all three cases—even the non-ideal sample re-thawed five times in a two-month time period—the *N*-glycan structures remained intact and could be analyzed. Thus, *N*-glycans derived in this way can be considered as good indicators of disease-related change identification from blood. Although during the further investigation different collection tubes were used at DRC, based on the obtained results, the preferred blood-collection tubes for *N*-glycan analysis should be the native tube, because in that case, most sialylated structures remained intact.

**Figure 2:**
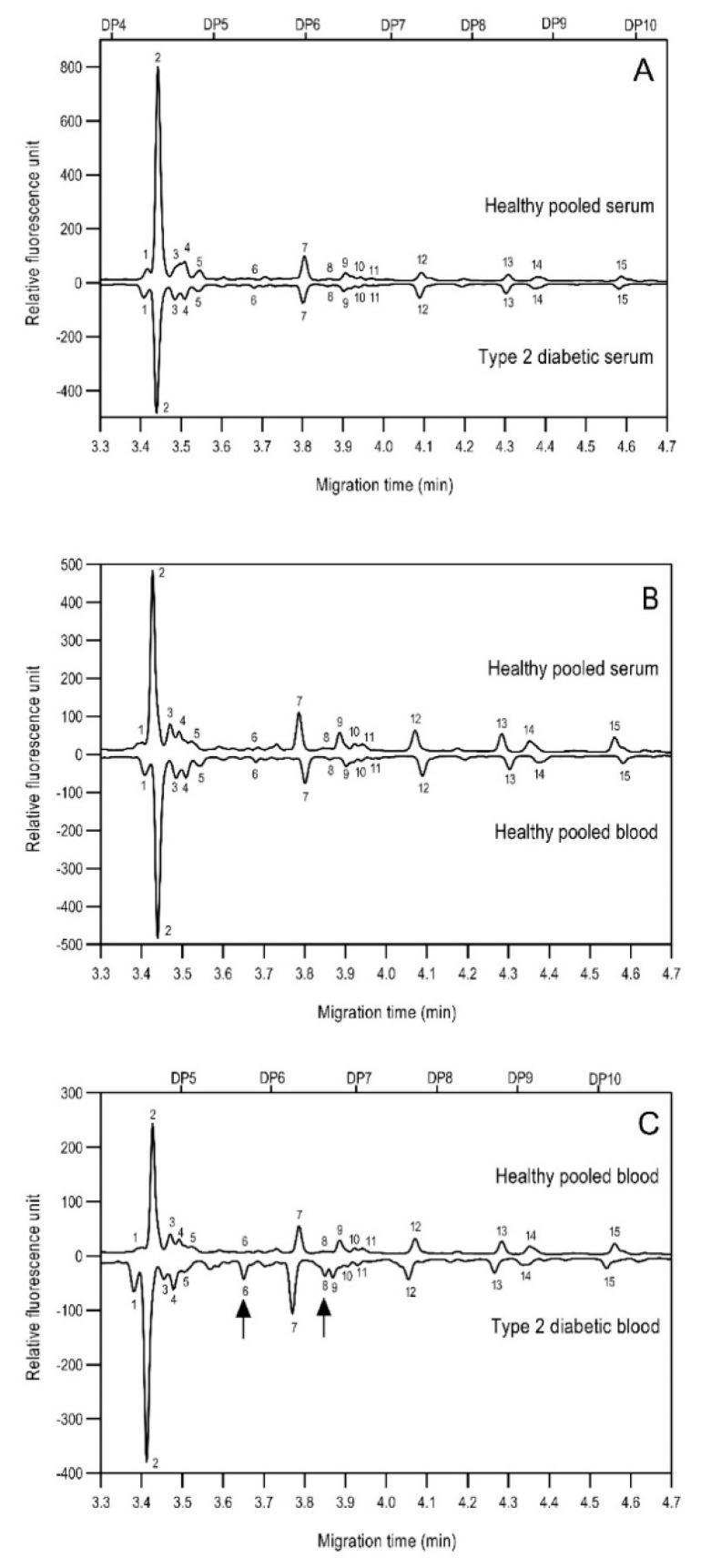
Comparison of the *N*-glycome of healthy and type 2 diabetic serum and blood samples. Panel (**A**)—healthy pooled vs. T2 diabetic serum sample, Panel (**B**)—healthy blood vs. healthy serum sample, (**C**)—healthy pooled vs. T2 diabetic blood sample with the indication of potential biomarker peaks by the arrows. Separation conditions were the same as in Figure 1. Structures corresponding to peaks: 1: FA4BG4S4, 2: A2G2S2, 3: A2BG2S2, M3, 4: FA2G2S2, 5: FA2BG2S2, 6: FA2G1S1, 7: A2G2S1, 8: A2BG2S1, 9: FA2G2S1, 10: FA2BG2S1, 11: A4G4S2, 12: FA2, M6, 13: FA2[6]G1, M7, 14: FA2[3]G1, FA2BG1, M8, 15: FA2G2.

As the next step, two types of liquid biopsy specimens were compared, serum and total blood. The analyses of the serum (pooled healthy and treated diabetic) and the total blood (pooled healthy and treated/untreated diabetic) were carried out systematically, as shown in Figure 2. First, the healthy and the treated T2D serum samples were compared (Figure 2, Panel A), where no apparent differences were found between the traces. Next, the serum and the whole blood glycosylation profiles were compared (Figure 2, Panel B) where, except for minor migration changes and peak-area alterations, no significant differences were observed. Finally, pooled healthy whole blood and the T2D whole blood glycosylation were compared (Figure 2, Panel C), in which instance two extra peaks (Peaks 6 and 8), appeared in the trace of the latter. Based on these results, peaks 6 and 8 *N*-glycans could be considered as potential biomarkers for T2D. Please note that, considering the results shown in Figure 1, all samples were collected using the K3E EDTA blood-collection tubes. The structural elucidation of serum *N*-glycans was reported earlier [7] and used in this study. Briefly, the structure identification utilized the direct mining of GU database entries (GUcal application linked with the GlycoStore data collection), and some earlier published literature data on similar samples [30,31]. Moreover, the glycan structure abbreviations followed the nomenclature of Harvey et al. [32]. The apparent minor-peak migration-time shifts in Figure 2 panel B and C were less than 0.5%, which is well below the acceptable value. The identified glycan structures are shown in Table 1, where each symbol represents a single monosaccharide and includes the positional and linkage information.

**Figure 3:**
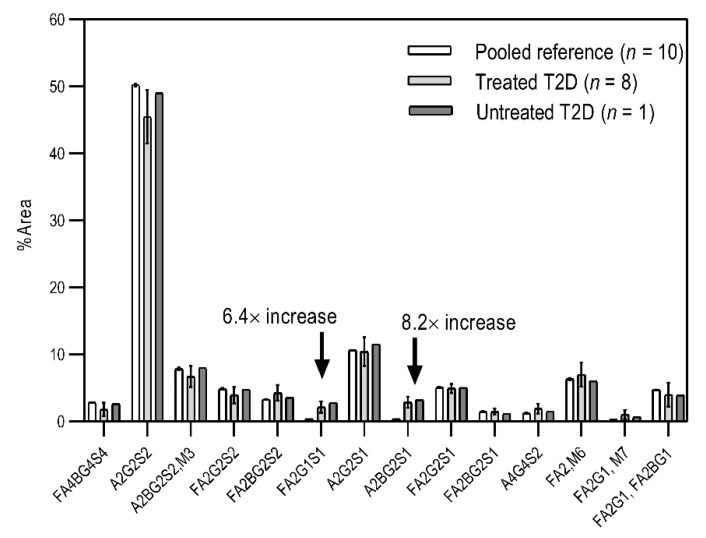
Comparison of the *N*-glycosylation of healthy (*n* = 10), treated T2D (*n* = 8) and untreated T2D (*n* = 1) blood samples. Separation conditions were the same as in Figure 1.

Based on the scouting results shown in Figure 2, additional T2D patients were involved in the study for a deeper analysis of the observed phenomena. From a single day, nine blood samples were collected from volunteers with T2D of which eight were already treated with HMG-CoA reductase inhibitor drugs, and one individual was untreated. Of the nine people, 60% were male with ages between 30 and 50 years. As a reference, the blood samples pooled 10 volunteers. In the case of the reference sample, the age of the reference group ranged between 30 and 40 years, with equally distributed genders. It is important to note that the type 2 diabetic blood samples were collected in K3E EDTA tubes, which showed different intensity % area in their *N*-glycosylation profile, as seen in the preliminary experiments using K2E EDTA tubes (shown in Figure 1). Thus, it was shown that it was highly important in this type of analytical approach to use the same type of tubes over time, to ensure the comparability of the obtained results. Each sample was prepared and analyzed similarly to those shown in Figure 2. The resulting peaks were integrated, averaged throughout all collected samples, and compared to the pooled reference sample, as shown in Figure 3. The figure includes the error bars which depict the standard deviation of the mean of all nine T2 diabetic samples measurements.

Although the glycosylation profiles of blood from healthy and T2D patients did not differ significantly, the relative amounts of the two formerly identified glycan structures increased in all nine cases, regardless of the treatment, showing a 6.4× and 8.2× increase in the concentrations of FA2G1S1 and A2BG2S1, respectively. Our results suggested that FA2G1S1 and A2BG2S1 *N*-glycans could be used as potential biomarkers for T2D.

## 3. Discussion

In this study, we reported on a simple capillary electrophoresis-based technique for *N*-glycosylation analysis of type 2 diabetic (T2D) patients using whole blood samples. First, the blood collection, storage, and handling conditions were investigated, as was their effect on the *N*-glycan profile. The sample handling included multiple instances of thawing and re-freezing, and three types of collection tubes. Based on the results, the *N*-glycan profiles remained similar, despite the applied artificial non-ideal conditions in which the whole blood samples were thawed and refrozen five times. Moreover, it was shown that the type of collection tube significantly influenced the resulting *N*-glycosylation profile. Based on our results, the recommended blood collection tube for *N*-glycosylation analysis should be the native type, because in that case most sialylated structures were preserved. Then, the *N*-glycan profiles of human serum and whole blood samples were compared between healthy individuals and T2D patients. The asparagine-linked carbohydrates were enzymatically released, fluorophore-labeled, and analyzed by capillary electrophoresis with high-sensitivity laser-induced fluorescence detection. Fifteen *N*-glycan structures were identified based on their glucose unit (GU) values. In the serum analysis, negligible profile differences were observed between the examined groups. Furthermore, the differences between the healthy serum and the T2D serum, as well as between the healthy serum and the healthy blood samples, were not significant. At the same time, our results showed that the whole blood profile changed significantly, exhibited by a significant increase in the concentrations of the FA2G1S1 and A2BG2S1 glycans (6.4× and 8.2×, respectively) in the case of T2D. Based on the two newly identified potential biomarkers, it is suggested that study samples from a larger cohort of patients be obtained to perform statistical analysis. In addition, information on the specific IgG *N*-glycosylation profiles in T2D could shed light on underlying inflammatory pathophysiological processes and could lead to drug targets.

## 4. Materials and Methods

### 4.1. Chemicals and Reagents

Water (HPLC-grade), acetonitrile, dithiothreitol (DTT), ammonium acetate, acetic acid, sodium cyanoborohydride (1 M in THF), glycerol, sodium dodecyl sulfate (SDS), and Nonidet P-40 (NP-40) were obtained from Sigma Aldrich (St. Louis, MO, USA). Aminopyrene-1,3,6-trisulfonic acid (APTS), the high-resolution *N*-linked carbohydrate separation buffer (HR-NCHO), the bracketing standard and the magnetic beads were part of the Fast Glycan Kit (Sciex, Brea, CA, USA). The human serum and blood samples were kindly provided by the Drug Research Center (Balatonfüred, Hungary). PNGase F enzyme was produced in-house (University of Pannonia, Veszprém, Hungary).

### 4.2. N-Glycan Sample Preparation from Blood

The samples were collected individually into four types of blood collection tubes: anticoagulant-free, K2E EDTA, K3E EDTA, and blood coagulation activator. The reference serum and blood samples from healthy individuals were pooled in order to minimize the information loss of species below the detection threshold, and to improve the efficiency following the method described in [34]. The sample preparation of the human blood and serum started by denaturation, endoglycosidase-based *N*-glycan release, APTS fluorophore labeling, and magnetic-bead-mediated cleanup. First, the blood samples were diluted to 50× by adding 2.0 µL blood sample into 98 µL of HPLC-grade water. Then, the samples were denatured at 80 °C for 10 min in a heating block, by adding 2.0 µL of denaturation mixture of 0.6% SDS, 12.5 mM DTT and 0.06% NP40. The *N*-glycans were then released from the denatured proteins by adding 1.0 µL of PNGase F in 20 µL of 18 mM ammonium acetate buffer (pH = 7.0) followed by incubation at 37 °C for 60 min. Then, evaporative fluorescent labeling was performed using 4.0 µL of 40 mM APTS in 20% acetic acid, 2.0 µL of NaBH_3_CN (1 M in THF), and 4.0 µL 20% acetic acid. The reaction mixture was incubated in a heating block with an opened cap at 37 °C overnight under a fume hood [35]. After the labeling step, the excess dye was removed by magnetic beads following the Fast Glycan Sample Preparation and Analysis protocol (Sciex) [36,37] and the APTS-labeled glycan samples were analyzed by CE–LIF.

### 4.3. Capillary Electrophoresis

Capillary electrophoresis analyses with laser-induced fluorescent detection (CE–LIF) (excitation: 488 nm, emission: 520 nm) were performed using a PA 800 plus Pharmaceutical Analysis System (Sciex). A 30 cm total (20 cm effective) length, 50 µm i.d. bare-fused silica capillary was used filled with HR-NCHO gel buffer (Sciex). Separation voltage was set to 30 kV (0.5 min ramp) in reversed-polarity mode (cathode at the injection side, anode at the detection side). The capillary temperature was set to 20 °C. The sample was introduced using a pre-injection as a water plug (5.0 psi for 5 s) followed by 6.0 kV for 3 s electrokinetic sample injection. Data acquisitions were performed by the 32Karat (version 10.1) software package (Sciex). The data evaluation, including peak areas and % area determination, was performed by CHROMuLAN (PiKRON s.r.o., Praha, Czech Republic). GU value calculation and structural *N*-glycan assignment were accomplished by using the GUcal software version 1.1c (University of Pannonia, Veszprém, Hungary) and its built-in database (www.gucal.hu—accessed on 16 August 2021) [30].

### 4.4. Clinical Patient Information

A total of 21 blood samples were collected from 10 healthy volunteers and 11 T2D patients of which 8 persons were already treated with HMG-CoA reductase inhibitor drugs, and 1 individual was untreated. All the donors were Caucasian, with the average age of 40.9, and a median age of 34, between 11 females and 10 males. All the samples were collected with the appropriate Ethical Permissions (approval number: 22051-3/2013/EKU (278/2013 and Informed Patient Consents at the DRC Drug Research Center (Balatonfüred, Hungary).

## Figures and Tables

**Table 1:** Identified *N*-glycan structures (nomenclature followed the guidelines of the Consortium of Functional Glycomics) [33]. Entry numbers correspond to the peak numbers in the Figures. Symbols: 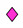 Sialic acid (*N*-acetylneuraminic acid); 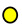 Galactose; 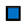 *N*-acetylglucosamine; 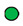 Mannose; 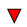 Fucose.

Peak No.	Peak ID	Structure
1	FA4BG4S4	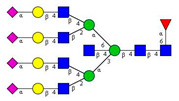
2	A2G2S2	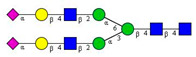
3	A2BG2S2; M3	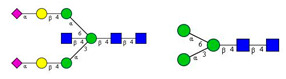
4	FA2G2S2	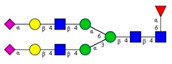
5	FA2BG2S2	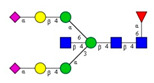
6	FA2G1S1	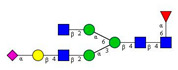
7	A2G2S1	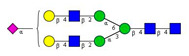
8	A2BG2S1	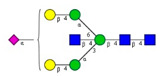
9	FA2G2S1	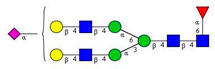
10	FA2BG2S1	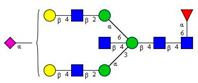
11	A4G4S2	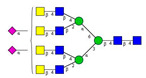
12	FA2; M6	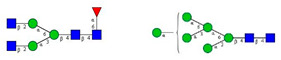
13	FA2[6]G1; M7	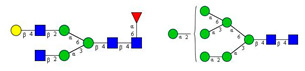
14	FA2[3]G1; FA2BG1	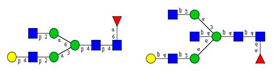
15	FA2G2	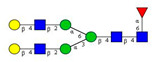

## Data Availability

All the relevant data are published in the paper.

## Sample Availability

Samples of the compounds are not available from the authors due to ethical reasons.
